# The Main Advantages of Community Based Participatory Health Programs: An Experience from the Islamic Republic of Iran

**DOI:** 10.5539/gjhs.v5n3p28

**Published:** 2013-01-21

**Authors:** Monir Baradarn Eftekhari, Katayoun Falahat, Masoumeh Dejman, Ameneh Setareh Forouzan, Hossein Malek Afzali, Noot Heidari, Arash Mirabzadeh

**Affiliations:** 1 Undersecretary for Research and Technology, Ministry of Health and Medical Education, Tehran, Iran; 2 Social Determinants of Health Research Center, Welfare and Rehabilitation University of Medical Science, Tehran, Iran; 3 School of Public Health, Tehran University of Medical Sciences, Tehran, Iran

**Keywords:** empowerment, qualitative study, assessment, community based participatory program, health, Iran

## Abstract

**Introduction:**

Community based participatory program is an approach that emphasize on community empowerment as an important tool in health promotion especially in low and middle income countries. The aim of this study is in-depth understanding the strengths point of active community based participatory programs in Iran in order to develop a scientific foundation and basis for future policy and decision making.

**Methods:**

This study was a qualitative study using focus group discussions. Thirteen community based programs related to health that were active for last five years were selected and assessed. Data analysis was based on deductive-inductive content analysis approach considering the predetermined structure according to study questions.

**Results:**

In this study, strengths points of community participatory health programs based on the locality of the implementation of the programs; governmental organization and nongovernmental organizations (NGO’s) were evaluated. The main strengths of these programs were the presence of the spirit of empathy and high motivation in working for community, absorbing the community assistance, community empowerment, presence of female volunteers, using local volunteers, creation of social prestige and evidence based decision making for community problem solving.

**Conclusion:**

Capacity building of the community, NGOs and policymakers plays key role in participation mechanisms, partnership, team working and mobilizing of necessary resources in the promotion of participatory community based health programs.

## 1. Introduction

Community based participatory program is an approach that emphasize on community empowerment as an important tool in health promotion (O’Toole, Aaron, Chin, Horowitz, & Tyson, 2003; Freudenberg, Pastor, & Israel, 2011). In this method, education and social action resulting to better use of health care services, improve health literacy and reduce health disparities (Wallerstein & Duran, 2006). Community based participatory program requires action at all levels of society and can only be possible through effective public participation. In fact, this approach has endeavored in order that, with the community’s participation, will be able to help them identify their problems and in this way be committed actively to take steps in solving problems by themselves, and as a consequence, would result the reduction of inappropriate healthy behaviors within the community (Amering, Stastny, & Hopper, 2005). In 2003, a study conducted by the University of California aiming at evaluating the community based participatory research have showed that this method increase understanding of complex community health problems, insure the policy and practice relevance of findings, and assist in using those findings to health promotion and prevent disease (Leung, Yen, & Minkler, 2004).

Also, in 2005, a study conducted in Michigan school of public health showed that using partnerships in CBPR enhance our understanding and addressing the multiple determinants of children’s health (Israel et al., 2005). In Iran, despite the antiquity and multiplicity of community based programs in public and private sectors, but unfortunately, extensive studies specifically regarding comprehensive evaluation of these programs have never been implemented or published. In 2009, Tabriz Health Management Center has conducted a study to assess the relationship between educational collaborative approach of the female health volunteers and their role in identifying the community health needs and this center has also planned to conduct more studies using female health volunteers (Behdjat, Rifkin, Tarin, & Sheikh, 2009). Also, in the year 2010, Iranian National Health Research Center has conducted a comprehensive study to assess the needs and set the priorities in one of deprived areas. The result of this study led to develop an operational plan for interventions (Mohammadi et al., 2010). Due to the disperse and limited studies on this subject matter, we have decided to conduct a comprehensive qualitative assessment of the strengths, in the community based health programs implemented in Iran with the hope that the results of this study, would encourage all researchers to do these projects to cover the challenges and gaps in existing information for the policymakers and managers. The aim of this study is in-depth understanding the strengths point of active community based participatory programs in Iran in order to develop a scientific foundation and basis for future policy and decision making.

## 2. Methods

This study was designed and implemented in a qualitative method. In this study, all governmental and nongovernmental community based participatory health programs which were actively involved during the past 5 years (2006-2010) were selected (13 programs) (Table 1). The number of eight program were affiliated to ministry of health and medical education, three program were non affiliated to this ministry and two programs were from non-governmental organization. Data were collected from September to March 2010 respectively. In this study, we employed the focus group discussion for the program volunteers (who were chosen from the community and work voluntarily) and the services recipients. The reason of selecting this method was high ability to explore people’s opinions, concerns, attitudes and experiences of individuals regarding a specific subject matter (R. Barbour & Kitzinger, 1999; Dahlgren, Emmelin, & Winkvist, 2007).

**Table 1:** Selected thirteen community based health programs in Iran

Governmental		Non-Governmental

Affiliated to The Ministry of Health & Medical Education	Non affiliated to the Ministry of Health & Medical Education	
Behvarzi	Laborious Health House program	Addiction control and prevention-Aftab Population
Population lab program	Community Based Rehabilitation	Tavana
Safe Society program	Municipality Health House program	
Campaign of Polio Eradication		
Women Health Volunteers program		
School Health (Pupil Volunteers)		
Healthy Village program		
Healthy City program		

The inclusion criteria for the volunteers was at least one year experience as a volunteer in a program, and for service recipients having a family health recorded profile in one of the health centers in their vicinity. Interviews were conducted on the areas of program implementation namely; Tehran, Qazvin and Saveh. To perform interviews, guide questionnaires of the focus group discussion were developed. These guide questionnaires was designed based on the following: the opinions of the research team and experts familiar with community based participatory programs, existing resources and objectives of the study. Themes being assessed in this guide questionnaire include main advantages of participatory based programs. Validity and reliability of the guide questionnaires were confirmed by a pilot study performed in a governmental program and a nongovernmental program by research team members, and then necessary modifications in sequence and timing of the guide questionnaires were carried out. To implement the study, representative of each program was informed of study objectives and their responsibility of coordinating and conducting focus group discussions. The number of focus group discussion was based on data saturation and a total of 20 group discussions were conducted. The number of people involved in this study is 140 in focus group discussions; consist of eighty service recipients and sixty program volunteers. Duration of each focus group discussions was at least 90 minutes and continued until achievement of data saturation. In each interview, the presence of a facilitator and an observer were strictly considered. Research team consisted of 6 members who were all experts in performing qualitative research and interviews. They were informed with the guide questionnaires as well as the objectives of the study in a training session. Each interview, with the permission of the participants, was recorded with the use of a tape recorder and transcribed at the end of each session.

### 2.1 Data Analysis

Data analysis was manually done using the inductive deductive content analysis method based on the study objectives. Initially, each tape recorded interviews were transcribed. Transcripts were read and then coded. In the next phase, codes that had similar concepts were categorized and were conceptually classified based on advantages of these programs. Then the extracted concepts were divided into 2 sections based on the locality of the implementation of the programs; governmental organizations (affiliated with the Ministry of Health and not affiliated with the Ministry of Health) and nongovernmental organizations (NGO’s).

In order to ensure the reliability and validity of the data, triangulation method was performed using combined group interviews from different participants including the recipients and volunteers. To address conformability, summarized interview findings was shared with the participants at the end of the group discussion (respondent validation) to get participants’ recognition of the finding. Peer checking by experienced colleagues to re-analyze some of the data was performed to assess dependability. Consistency checks between colleagues were also performed throughout the coding process (team consistency) (R. S. Barbour, 2001; Rolfe, 2006).

For the purpose of being able to transfer the data, a research team member was present in the field of study and the details of the method of the study was evaluated and compared by the observer/supervisor.

### 2.2 Ethical Considerations

Ethical considerations including the acquisition of informed consent from all participants prior to the study, maintaining the anonymity of the participants, confidentiality of information and the right to withdraw from the study were considered. Also, the result of the study was sent to all stakeholders.

## 3. Result

In this study, strengths points of community participatory health programs based on the locality of the implementation of the programs as governmental organization (affiliated with the Ministry of Health and not affiliated with the Ministry of Health) and nongovernmental organizations (NGO’s) were presented as follow

### 3.1 Presence of the Spirit of Empathy and High Motivation in Working for Community

In both of governmental and nongovernmental programs, presence of the spirit of empathy and high motivation in working for community has been pointed as the strongest point by the majority of the programs participants especially in the initiation phase.

“We like people and we want to do for them something ”

### 3.2 Evidence Based Decision Making in Community Problem Solving

Some participants especially in nongovernmental programs believed that in these programs, decision making process is based on the needs of the community and implemented from the bottom to the top level. However, in some programs, existing of basic structure in system and the possibility of using existing structures in program improvement were considered as the advantages of them.

“When you ask us about our problems, I think, they will be solved certainly…”

### 3.3 Absorbing the Community Assistance

Community mobilization and absorbing the community assistance, high spirit of generosity and humanitarian assistance through charitable individuals’ and the fact that nongovernmental community based programs are independent in terms of financial aspects, the perseverance and good deliberation of stakeholders have been pointed as the strong points of the nongovernmental programs.

### 3.4 Presence of Female Volunteers

The volunteers’ high morale and strong motivation in delivering services is considered the most important strong point in these programs. The presence of female volunteers especially in the governmental programs has given strength to the programs, as pointed by the majority of service recipients.

“The utilization of the women’s wasted time especially the housewives and converting it productively in line with the program has become the strong point of the program”

### 3.5 The Use of the Local Volunteers

The use of the local volunteers particularly in the rural areas is also another important strong point of the program as pointed out by the majority of the participants of the nongovernmental programs. Services recipients trust with no anxiety of being judged and considering clients’ self esteem and independency were expressed as strong points by the participants from nongovernmental programs.

### 3.6 Creation of Social Prestige

Based on the opinions of the majority of the volunteers, the creation of social prestige and recognizing women in community and increasing their self-confidence is one of the most important strong points in programs especially in Women Health Volunteers program. Building capacity for women in the volunteers’ point of view is “prestige and social standing” while the services recipients believed that this has been the main factor which increases their knowledge and awareness.

### 3.7 Community Empowerment

Based on the beliefs of all participants, capacity building is the major strong point in all participatory programs particularly on its initial period. Majority of participants have pointed out that one way of achieving the goals of the health systems is through building capacities of community that result in community high participation in order to solve their problems. In fact all participants’ believed that increasing the awareness and health education at the community level especially among women is an effective factor in promoting community based participatory programs. Change of attitudes and behavior modifications as a sequence of increasing awareness have also been addressed by program volunteers in some governmental programs while services recipients of nongovernmental programs believed that capacity building programs has been designed based on their needs and are up to date. Table 2 illustrated the main strengths of community based participatory program based on affiliated to GOs /NGOs.

**Table 2:** The strengths points of community based participatory program based on affiliated to GOs /NGOs

Strengths’ points	Governmental program	Nongovernmental program
Presence of the spirit of empathy and high motivation in working for community	✓ +	✓ +
Evidence based decision making in community problem solving	✓	✓ +
Absorbing the community assistance	✓	✓
Presence of female volunteers	✓ +	-
The use of the local volunteers	✓ +	✓
Creation of social prestige	✓ +	-
Community empowerment	✓ +	✓

✓ +: The most ✓ : More - : The least

## 4. Discussion

According to the results of the study, the majority of the strength points in the community based participatory programs assessed are the volunteers’ high spirit of empathy and strong motivation in providing their services. In this sense, the cooperation and coordination of the women have particularly played a vital role in the advancement of all the programs. On the other hand, the community’s capacity building processes (bottom to top process) were also found to be the other strong point of most community based participatory programs.

In this study, the presence of female volunteers in the community based participatory programs has played a key role in the advancement of the program. Basing on the objectives of the United Nations Millennium Development Goals, building the capacities of women has been proven to be very effective in promoting gender equity and considered one of the key strategies in development (Clemens, Kenny, & Moss, 2007). On the other hand, women’s capacity building in the different areas of health has played a vital role in the promotion health literacy in the society (Marmot, Friel, Bell, Houweling, & Taylor, 2008). The study done by Gutierrez in 2005 has shown the significant role of women in grassroots organizations and has shown that women are usually more interested in volunteer works than their male counterparts (Minkler, 2004; Ziersch, Osborne, & Baum 2011). The process of decision making has been considered as one of the strengths point seen in some programs, but in other programs, this process is not completely based on evidence and the different stakeholders don’t cooperate for needs assessment and priority setting and this is a weak point of them. The results of study conducted by the World Health Organization in different countries worldwide in such a way that the Commission on the Social Determinants of Health which is under the supervision of the WHO has issued a notification to all countries in order to considering the participation of all stakeholders in the decision making process (Barten, Mitlin, Mulholland, Hardoy, & Stern, 2007).

The presence of institutionalization of some programs in the health system such as behvarzi not only creates reform and update the programs but also resolve obstacles.

Financial independency of nongovernmental affiliated programs to government`s budgets can increase absorbing community assistance. In some countries including Brazil, the factors that contribute in the success of community based participatory programs are financial support and the community’s active participation in budget categorization (Barten et al., 2007).

## 5. Limitations

The present study illustrates the opinions of the people in the 20 focus groups discussions and should for methodological reasons (qualitative approach) not be generalized to other condition. Also, lack of participation of male volunteers and service users in group discussions was another limitation in this study.

## 6. Conclusions

It is important to note that the process of building capacities of the community using the participatory approaches has not only resulted in an increase in the community’s capacities but also played an important factor in improvement and development of participatory programs as one of the key strategy in achieving effectiveness in health equity. The result of community based process assessment as the main component of this approach can help national policy makers to do more support from these programs in order to disseminate evidence based decision making. Also, our findings may be useful for institutionalizing the community based participatory programs in health system. Finally, cooperation of private sectors and nongovernmental organizations can develop these programs and facilitate the community mobilization.
